# Association Between Progesterone Elevation on the Day of Human Chronic Gonadotropin Trigger and Pregnancy Outcomes After Fresh Embryo Transfer in *In Vitro* Fertilization/Intracytoplasmic Sperm Injection Cycles

**DOI:** 10.3389/fendo.2018.00201

**Published:** 2018-04-26

**Authors:** Sandro C. Esteves, Gautam Khastgir, Jatin Shah, Kshitiz Murdia, Shweta Mittal Gupta, Durga G. Rao, Soumyaroop Dash, Kundan Ingale, Milind Patil, Kunji Moideen, Priti Thakor, Pavitra Dewda

**Affiliations:** ^1^ANDROFERT, Andrology and Human Reproduction Clinic, Campinas, Brazil; ^2^Division of Urology, Department of Surgery, Faculty of Medical Sciences, University of Campinas (UNICAMP), Campinas, Brazil; ^3^Faculty of Health, Aarhus University, Aarhus, Denmark; ^4^BIRTH, Kolkata, India; ^5^Mumbai Fertility Center – Kamala Polyclinic & Nursing Home, Mumbai, India; ^6^Indira IVF Group, Udaipur, India; ^7^Sir Ganga Ram Kolmet Hospital, New Delhi, India; ^8^Oasis Centre for Reproductive Medicine, Hyderabad, India; ^9^Srishti Assisted Fertility & Advanced Laparoscopy Center, Srishti Hospital, Moolakulam, India; ^10^Nirmiti Clinic, Centre for Assisted Reproduction, Chinchwad, India; ^11^Shobha Nursing Home, Solapur, India; ^12^ARMC IVF Fertility Centre, Kozhikode, India; ^13^Merck Limited, Mumbai, India

**Keywords:** assisted reproductive technology, controlled ovarian stimulation, human chorionic gonadotropin trigger, intracytoplasmic sperm injection, *in vitro* fertilization, late follicular phase, pregnancy outcomes, progesterone elevation

## Abstract

Progesterone elevation (PE) during the late follicular phase of controlled ovarian stimulation in fresh embryo transfer *in vitro* fertilization (IVF)/intracytoplasmic sperm injection cycles has been claimed to be associated with decreased pregnancy rates. However, the evidence is not unequivocal, and clinicians still have questions about the clinical validity of measuring P levels during the follicular phase of stimulated cycles. We reviewed the existing literature aimed at answering four relevant clinical questions, namely (i) Is gonadotropin type associated with PE during the follicular phase of stimulated cycles? (ii) Is PE on the day of human chorionic gonadotropin (hCG) associated with negative fresh embryo transfer IVF/intracytoplasmic sperm injection (ICSI) cycles outcomes in all patient subgroups? (iii) Which P thresholds are best to identify patients at risk of implantation failure due to PE in a fresh embryo transfer? and (iv) Should a freeze all policy be adopted in all the cycles with PE on the day of hCG? The existing evidence indicates that late follicular phase progesterone rise in gonadotropin releasing analog cycles is mainly caused by the supraphysiological stimulation of granulosa cells with exogenous follicle-stimulating hormone. Yet, the type of gonadotropin used for stimulation seems to play no significant role on progesterone levels at the end of stimulation. Furthermore, PE is not a universal phenomenon with evidence indicating that its detrimental consequences on pregnancy outcomes do not affect all patient populations equally. Patients with high ovarian response to control ovarian stimulation are more prone to exhibit PE at the late follicular phase. However, in studies showing an overall detrimental effect of PE on pregnancy rates, the adverse effect of PE on endometrial receptivity seems to be offset, at least in part, by the availability of good quality embryo for transfer in women with a high ovarian response. Given the limitations of the currently available assays to measure progesterone at low ranges, caution should be applied to adopt specific cutoff values above which the effect of progesterone rise could be considered detrimental and to recommend “freeze-all” based solely on pre-defined cutoff points.

## Introduction

Progesterone (P) is essential before and during pregnancy as it plays a critical role in supporting the endometrium and hence survival of the conceptus (1). In the natural cycle, preovulatory P secretion facilitates the action of estrogen on the pituitary; the latter is the key factor to induce the mid-cycle luteinizing hormone (LH) peak. Progesterone also stimulates the mid-cycle follicle-stimulating hormone (FSH) surge, which is important to support the expression of LH receptors in the granulosa layer (2, 3). Notably, most circulating P (~95%) is produced in the intrafollicular compartment by the granulosa cells (GCs) *via* the action of 3β-HSD that catalyzes the conversion of pregnenolone (delta-4 pathway) under LH influence (4, 5). After ovulation, the corpus luteum is formed and P is produced in both luteinized theca and GCs under the effect of endogenous LH activity (6). In early pregnancy, human chorionic gonadotropin (hCG) secreted by syncytio trophoblast cells rescues the corpus luteum and maintains luteal function until placental steroidogenesis is established (7). As such, progesterone elevation (PE) and its sustained levels have been considered essential to elicit the endocrine signals responsible for initiating the period of endometrial receptivity to embryo implantation (8, 9).

Late follicular phase PE, commonly defined as P levels of 1.5 ng/ml (4.77 nmol/l) or greater at the day of hCG trigger, has been reported in 6–30% of controlled ovarian stimulation (COS) cycles (10–14). The observation of worse pregnancy outcomes in fresh embryo transfer *in vitro* fertilization (IVF)/intracytoplasmic sperm injection (ICSI) cycles among patients with PE compared to non-PE has prompted clinicians to monitor progesterone levels during the late follicular phase or at the day of hCG trigger. A policy of freezing all embryos from a fresh IVF/ICSI cycle and replacing the embryos in a subsequent cycle has been advocated as a solution to avoid the potential negative effect of PE on pregnancy (15, 16).

In non-gonadotropin releasing hormone (GnRH) analog cycles, premature PE can be explained by an early preovulatory LH elevation (17, 18), which results in endometrial asynchrony that ultimately affects implantation and pregnancy (19). In contrast, follicular phase PE cannot be attributed to premature LH surge in GnRH analog cycles, since the pituitary is suppressed (20–24). Furthermore, whereas some investigators have reported an inverse association between pregnancy rates in fresh embryo transfer IVF/ICSI cycles and PE, the evidence is not unequivocal thus making the universal application of the freeze-all policy debatable (11).

Currently, many clinicians have questions about the clinical validity of measuring P levels during the follicular phase of stimulated cycles. Among several concerns, it is not clear which patients might benefit from P monitoring and what would be the practical implications of PE to pregnancy success in an IVF/ICSI program. In this review, we summarize the recent evidence concerning the clinical implications of PE on Assisted Reproductive Technology (ART) cycles outcomes and identify gaps of knowledge as well as opportunities for future research.

## Review Criteria

### Search Criteria

An extensive search of studies published in the past 10 years by examining the relationship between P levels on the day of hCG trigger and pregnancy outcomes in fresh embryo transfer IVF/ICSI cycles was performed using PubMed and MEDLINE. The start and end dates for the searches were January 2006 and February 2017, respectively.

### Eligibility Criteria

Specifically, our study was designed to answer clinical questions raised by the group of authors with clinical experience in IVF/ICSI (SE, GK, JS, KM, SG, DR, SD, KI, and MP), namely (i) Is gonadotropin type associated with PE during the follicular phase of stimulated cycles? (ii) Is PE on the day of hCG associated with negative fresh embryo transfer IVF/ICSI outcomes in all patient subgroups? (iii) Which P thresholds are best to identify patients at risk of implantation failure due to PE in a fresh embryo transfer? and (iv) Should a freeze all policy be adopted in all the cycles with PE on the day of hCG? Therefore, eligible studies were those that provided evidence to answer one or more questions above.

### Study Selection

The overall strategy for study identification and data extraction was based on the following key words: “assisted reproductive technology,” “controlled ovarian stimulation,” “intracytoplasmic sperm injection,” “*in vitro* fertilization,” “progesterone levels,” “hCG trigger,” “pregnancy outcomes,” with the filters “humans,” and “English language.” Data only published in conference or meeting proceedings, websites, or books were not included. Articles were only included if hCG trigger alone followed by conventional luteal phase support, which specifically means luteal supplementation with progesterone administration, and fresh embryo transfers were carried out. Oocyte donation and frozen-thawed embryo transfer cycles were excluded. Case reports and letters were not considered. Articles published before the start search date and book-chapter citations provided conceptual content only.

## Results and Discussion

The search retrieved a total of 31 articles that specifically addressed at least one of the formulated clinical questions. The Table S1 in Supplementary Material lists the eligible studies included to address the key questions.

### Is Gonadotropin Type Associated With PE During the Follicular Phase of Stimulated Cycles?

The menotropin versus recombinant FSH *IVF* trial (MERIT) compared to ongoing pregnancy rates (OPR) in 731 young, normogonadotrophic women undergoing IVF after stimulation with highly purified human menopausal gonadotrophin (HP-hMG; *n* = 363) or recombinant FSH (rFSH; *n* = 368) (25). The threshold value for defining serum PE in this study was 4 nmol/l (1.25 ng/ml), measured on the last day of stimulation. The serum P levels were higher in rFSH-treated patients than in HP-hMG-treated patients, with the former showing a higher incidence of PE (23%, 3.4 nmol/l versus 11%, 2.6 nmol/l; *p* < 0.001). In the study mentioned above, patients showing higher P values had lower fresh embryo implantation rate (HP-hMG group: 24 versus 19%, not statistically significant; rFSH group: 23 versus 11%, *p* = 0.025). The authors concluded that late follicular phase PE affected fresh embryo transfer IVF/ICSI outcomes, with PE being more common with the use of rFSH than HP-hMG, however, it is important to note that values of PE are below 4.77 nmol/l (1.5 ng/l) in both groups implying statistically significant values could be clinically irrelevant (25).

Contrary results were reported by Andersen et al. who performed a *post hoc* analysis of data from a randomized controlled trial (RCT) that included 475 women younger than 40 years subjected to IVF/ICSI (26). The primary aim of the study was to investigate whether the addition of recombinant LH (rLH) or FSH from day 6 of stimulation onward impacted pregnancy rates in GnRH agonist cycles. Moreover, the authors evaluated whether the addition of rLH in the second half of the follicular phase influenced serum P levels measured on the day of hCG trigger. P levels were determined on day 1, i.e., before exogenous FSH administration, and on the day of hCG trigger. Although patients receiving rLH had higher LH levels at the end of stimulation than those receiving rFSH alone, there were no differences in pregnancy rates and late follicular phase P levels between groups. In this study, P levels were associated with both the number of developing follicles and retrieved oocytes and partly to the late follicular phase LH concentration. Interestingly, higher pregnancy rates were reported in women with P concentration >7 nmol/l (>2.20 ng/ml) in the late follicular phase, which coincided with those developing high number of follicles (26).

In another study, Kolibianakis et al. (27) pooled data from five COS-IVF trials (28–32) using either GnRH antagonists or agonists that evaluated the impact of the type of gonadotropin, rFSH alone, rFSH combined with rLH, HP-hMG alone, and rFSH combined with HP-hMG on PE. The authors found that P levels in the late follicular phase were associated with the number of oocytes retrieved and serum estradiol levels, irrespective of type of GnRH analog, but not with the type of gonadotropin administered. Furthermore, there was no association between PE and duration of stimulation or FSH requirement. Subsequently, Requena et al. corroborated these aforementioned findings by showing that the mean serum P levels did not differ significantly with respect to the type of gonadotropin used for COS: rFSH + rLH (*n* = 377, P: 1.01 ng/ml), rFSH alone (*n* = 728, P: 1.06 ng/ml), rFSH + HP-hMG (*n* = 1,375; P: 1.30 ng/ml), and HP-hMG alone (*n* = 370; P: 1.10 ng/ml) (33). Last, a randomized, open-label, assessor-blind study comparing the efficacy and safety of HP-hMG and rFSH for COS reported that at the end of stimulation, the mean P levels were was not significantly different between HP-hMG (3.1 ± 3.4 nmol/l) and rFSH (3.1 ± 3.3 nmol/l) treatment groups (34). The characteristics and main findings of the studies mentioned above are summarized in Table 1.

**Table 1:** Characteristics of included studies to discuss if gonadotropin type is associated with progesterone elevation (PE) during the follicular phase of stimulated cycles.

Study and year (reference)	Patient characteristics	Ovarian stimulation regimen	Progesterone threshold	PE incidence
Menotrophin versus recombinant FSH (rFSH) *IVF* trial (MERIT) (25)	Study included a total of 731 young, normogonadotropic women undergoing IVF, patients were divided in two groups	Stimulation was performed with highly purified human menopausal gonadotropin (HP-hMG; *n* = 363) or rFSH (*n* = 368). P concentration was measured on the last day of stimulation	The threshold value for defining serum PE was 4 nmol/l (1.25 ng/ml)	The serum P levels were higher in rFSH-treated patients than in the HP-HMG-treated patients, with the former showing a higher incidence of PE (23 versus 11%; *p* < 0.001)

Andersen CY et al. (26)	Study included a total of 475 women age with 40 years, undergoing IVF or ICSI with a regular (21–35 days) menstrual cycle and basal serum FSH concentration of <10 IU/l on menstrual cycle day 2–5	Stimulation was performed with GnRH agonist and FSH in one group (*n* = 247), in another group (*n* = 228) patients were administered GnRH agonist, rFSH with rLH from day 6 of stimulation. P concentration was measured on day 1, prior to exogenous FSH administration, and on the day of hCG administration	The threshold value for defining serum PE was 4.77 nmol/l (1.5 ng/ml)	The average progesterone concentration on the day of ovulation induction (day of HCG administration) did not differ between those who received embryo transfer and those who did not [mean ± SD, 4.38 ± 3.90 nmol/l, (*n* = 419), versus 3.99 ± 2.16 nmol/l, (*n* = 56), respectively]

Requena et al. (33)	A total of 2,850 infertile women who were classified as high responders (high response was defined as women who had ≥20 oocytes retrieved or whose estradiol levels were ≥3,000 pg/ml) and were undergoing assisted reproduction techniques for the last 2 years were included in this retrospective study	Ovarian stimulation was performed by one of four possible methods: recombinant follicle stimulating hormone (rFSH) alone; rFSH combined with recombinant luteinizing hormone (rLH); highly purified human-menopausal gonadotropin (HP-hMG) alone; or rFSH combined with HP-hMG	The threshold value for defining serum PE was following in different groups: <0.5 ng/ml (<p10), 0.50–0.70 ng/ml (p10–p25), 0.71–1.00 ng/ml (p25–p50), 1.01–1.40 ng/ml (p50–p75), 1.41–1.80 ng/ml (p75–p90), and >1.81 ng/ml (>p90)	No significant differences in the mean progesterone concentration with respect to the type of gonadotropin that was used for ovarian stimulation: rFSH alone (*n* = 728, progesterone 1.06 ng/ml), rFSH + rLH (*n* = 377, progesterone 1.01 ng/ml), HP-hMG alone (*n* = 370; progesterone 1.10 ng/ml), and rFSH + HP-hMG (*n* = 1,375; progesterone 1.30 ng/ml)

Devroey et al. (34)	Study included women aged 21–34 years with a body mass index (BMI) of 18–25 kg/m^2^; primarydiagnosis of infertility being unexplained infertility or mild male factor; eligible for ICSI, infertile for12 months before randomization; with regular menstrual cycles of 24–35 days	Controlled ovarian stimulation was performed with HP-hMG or rFSH in a GnRH antagonist cycle with compulsory single-blastocyst transfer on day 5 in one fresh or subsequent frozen blastocyst replacement in natural cycles	The threshold value for defining serum PE was 3.18 nmol/l (1.0 ng/ml)	The average serum P level and the proportion of patients with serum P concentrations above 4 nmol/l at the end of stimulation (16% in the HP-hMG group and 14% in the rFSH group) were similar between the treatment groups

Lawrenz et al. (35)	ENGAGE study: a total of 1,506 women aged 18–36 years undergoing IVF stimulation cycles PURSUE study: a total of 1,390 women aged 35–42 years undergoing IVF stimulation cycles In both studies, women had a body weight of between 50 and 90 kg, regular menstrual cycles	For ENGAGE study: stimulation protocol included either a single injection of 150 mg CFA or daily injections of 200 IU rFSH in the first week of stimulation, using a standard GnRH antagonist protocol For PURSUE study: stimulation protocol included either a single injection of 150 mg of CFA or daily 300 IU of rFSH for the first week, again using a standard GnRH antagonist protocol. In both trials, daily rFSH was continued until three follicles reached >17 mm in size	The threshold value for defining serum PE was 1.5 ng/ml. PE was analyzed on day 8 of stimulation	Of patients with CFA, only stimulation 5.4% (13/239 patients) showed a PE above 1.5 ng/ml on day of hCG trigger, whereas patients with rFSH stimulation had a significant higher incidence of PE (18.3%; 62/339 patients) (*p* < 0.001)

Collectively, these findings suggest that gonadotropin type does not play a major role to PE in COS. During follicular phase, the GCs are supraphysiologically stimulated by gonadotropins, which may result in increased serum P levels (36). As the pituitary is normally suppressed by GnRH analogs during COS, the serum P levels in stimulated cycles represent the total follicle output of GCs (17, 33). The findings that LH activity provided by rLH of hCG content in hMG preparations is unable to influence P levels is explained by the fact that cytochrome 17a-hydroxylase-C17, 20 lyase (P450-17α), the key enzyme driving the conversion of intrafollicular P to estradiol, is virtually absent in the intrafollicular compartment (36–38), which makes the conversion of P to estradiol negligible in humans (39). PE is rather dependent on the overall GC output and is, therefore, associated with the number of follicles, oocytes, and E2 levels. Notwithstanding, some evidence suggests that the use of rFSH may be associated with higher P output than urinary gonadotropin normal and high responders, due to the higher potency of rFSH than urinary products (35, 40).

### Is PE on the Day of hCG Associated With Negative Fresh Embryo Transfer IVF-ICSI Outcomes in All Patient Subgroups?

In a systematic review and meta-analysis including 63 studies and over 60,000 IVF/ICSI cycles, Venetis et al. reported that late follicular phase PE was detrimental to fresh embryo transfer pregnancy rates. PE affected pregnancy success with levels as low as 0.8–1.1 ng/ml (OR: 0.79, 95% CI: 0.67–0.95), which increased as the level reaches 1.2 ng/ml (OR: 0.67, 95% CI: 0.53–0.84), and becomes stable thereafter (12). Along the same lines, Bosh et al. showed an association between P levels >1.5 ng/ml at the day of hCG administration and fresh embryo transfer IVF/ICSI pregnancy outcomes. The authors examined their database of 4,032 patients subjected to IVF/ICSI and found that OPRs were higher in patients with serum P levels ≤1.5 ng/ml than those with P levels >1.5 ng/ml (31.0 versus 19.1%; *P* = 0.00006) (10).

However, the evidence is not unequivocal as Miller et al. showed that the elevation of P level in 293 patients subjected to COS with hMG and/or FSH in a GnRH agonist protocol did not affect oocyte quality and pregnancy rate (41). The findings of Miller et al. were corroborated by Hamdine et al. who conducted a prospective intervention study including 158 IVF-ICSI patients (42). The authors showed that the incidence of PE (>1.5 ng/ml) was 13.3%, but OPRs were not significantly different between patients with normal P levels and PE (27.0 versus 19.0%). Furthermore, no differential impact of early or late GnRH antagonist initiation on the effect of elevated or normal P on OPR was observed. Likewise, the study of Andersen et al. mentioned above (26) reported that despite being strongly associated with the number of follicles and retrieved oocytes, late-follicular phase progesterone concentrations was not associated with clinical pregnancy rates (CPRs).

Recently, it has been suggested that the impact of PE on IVF/ICSI may vary according to the affected population. In one study, Griesinger and colleagues analyzed the data from six IVF/ICSI clinical trials to investigate the impact of P measured on the day of hCG trigger on fresh embryo transfer pregnancy outcomes, using the cutoff point of 1.5 ng/ml. Patients were stratified according to the number of oocytes retrieved after COS: low (1–5 oocytes), normal (6–18 oocytes), and high (>18 oocytes). The incidence of PE was 4.5 and 19.0% in low responders and high responders, respectively. Overall, OPRs per started cycle were significantly lower in women with PE [odds ratio (25): 0.55; 95% CI: 0.37–0.81]. However, a subgroup analysis showed that P level >1.5 ng/ml was associated with decreased pregnancy rates in low to normal responders, but not in high responders (11). In their study, OPRs in high responders with PE were higher than normal responder counterparts. In women without P elevation, the observed OPR increased from 29.9% (1–5 oocytes) to 39.2% (>18 oocytes). By contrast, women with elevated P showed an increase in OPR from 18.2% (1–5 oocytes) to 43.2% (>18 oocytes). Compared with the subjects without P elevation, the observed OPR were numerically lower in all subsets for the women with elevated P except for the high responders (>18 oocytes).

Similar results were reported by Requena et al. retrospectively analyzing the effect of PE on fresh embryo transfer IVF/ICSI pregnancy outcomes of 2,850 women classified as high responders (33). The high ovarian response was defined as having ≥20 oocytes or estradiol ≥3,000 pg/ml. The patients were subgrouped into six categories based on the level of serum P on day of hCG, as <0.5, 0.50–0.70, 0.71–1.00, 1.01–1.40, 1.41–1.80, and >1.81 ng/ml. The authors observed that P levels neither had a negative impact on the oocyte quality and endometrial receptivity nor did it affect pregnancy success. Only in the group of P level >1.80 ng/ml there was a marginally significant negative impact on pregnancy rates (OR: 0.73, 95% CI: 0.61 to 0.99) (33). Last, a retrospective analysis of 1,800 IVF/ICSI cycles performed by Cruz et al., who stratified patients by high (*P* > 1.5 ng/ml) or low (*P* < 1.5 ng/ml) concluded that PE in high ovarian responders did not impact fresh embryo transfer IVF/ICSI outcomes. The authors showed no significant differences in the analyzed parameters, namely, number of retrieved oocytes (17.2 ± 0.8 versus 17.3 ± 0.4), number of transferred embryos (1.81 ± 0.08 versus 1.85 ± 0.02), pregnancy rate (59.9 versus 54.6%), implantation rate (41.2 versus 39.7%), and miscarriage rate (22.6 versus 28.6%) for high and low progesterone levels respectively in case of high ovarian response (43).

It has been suggested that the likely reason for the negligible effect of PE on cycle outcome of high responders relates to the availability of high-quality embryos for transfer, including blastocysts, which could circumvent a possible adverse endometrial environment for implantation (42, 44). This hypothesis has been confirmed by the data of an early study by Papanikolaou et al., in which the impact of PE on pregnancy outcome was compared between day 3 and blastocyst single embryo transfers. The authors’ analyzed data from 482 patients undergoing single ET after COS with GnRH antagonist associated with rFSH. The incidence of PE (P above 1.5 ng/ml on the day of hCG administration) was 18.2%. The authors reported that PE did not affect pregnancy outcome in fresh blastocyst transfer cycles. In contrast, even a modest rise in P affected the pregnancy outcome in patients with day 3 embryo transfers (29).

In conclusion, there is conflicting data on the impact of late follicular phase PE on fresh embryo transfer IVF/ICSI outcomes. Although PE has been associated with decreased pregnancy rates in several studies, PE does not seem to affect all patient populations equally with high responders with PE achieving similar pregnancy success than counterparts without PE. In studies showing an overall detrimental effect of PE on pregnancy rates, the adverse effect of PE on endometrial receptivity seems to be offset, at least in part, by the availability of good quality embryo for transfer in women with a high ovarian response. In contrast, elevated P level results in reduced pregnancy in patients with a low ovarian response, who tends to have poorer quality embryos that counterpart with high ovarian response. Table 2 summarizes the studies discussed above.

**Table 2:** Characteristics of included studies to discuss if progesterone elevation (PE) on the day of hCG was associated with negative fresh embryo transfer IVF-ICSI outcomes in all patient subgroups.

Reference and place of study conducted	Design	Patient population	Intervention/method	Results
Venetis et al. (13) country: Greece	Retrospective analysis	A total of 3,296 women undergoing fresh IVF/ICSI	Simple bivariate analyses and multivariate analyses was done to compare PE and LBR according to serum P levels ≤1.5 versus >1.5 ng/ml on the day of HCG administration and compared among low (<6 oocytes), normal (6–18 oocytes), and high (>18 oocytes) responders	PE negatively impacted pregnancy success with levels as low as 0.8–1.1 ng/ml (OR: 0.79, 95% CI: 0.67–0.95). The magnitude of effect size increased as P level reached 1.2 ng/ml (OR: 0.67, 95% CI: 0.53–0.84), and became stable thereafter

Andersen et al. (26) country: Europe (22 centers): 10 in Denmark, 2 in Finland, 4 in Norway, and 6 in Sweden	Retrospective analysis	A total of 475 patients undergoing IVF/ICSI following ovarian stimulation with GnRH agonist and rFSH with or without rLH administration from day 6 of stimulation were included	The study was aimed to explore the association between the number of eggs and live birth outcomes, a likelihood logistic model was used to compare progesterone concentrations in two groups	Progesterone concentration was strongly associated with the number of follicles and retrieved oocytes. There was no significant association between the late-follicular phase progesterone concentration and clinical pregnancy rate

Griesinger et al. (11) country: meta analysis data collected from different study IVF centers in USA and Europe	Retrospective combined analysis	1,866 women undergoing IVF/ICSI with available serum P levels on the day of hCG	Univariate and multivariate analyses was done to assess association between elevated P level on the day of hCG, according to serum P levels ≤1.5 versus >1.5 ng/ml and compared among low (1–5 oocytes retrieved), normal (6–18 oocytes), and high (>18 oocytes) responders	The incidence of PE was 4.5 and 19.0% in low responders and high responders, respectively. Overall, OPRs per started cycle were significantly lower in women with PE (OR: 0.55; 95% CI: 0.37–0.81). However, a subgroup analysis showed that P level >1.5 ng/ml was associated with decreased pregnancy rates in low to normal responders, but not in high responders

Requena et al. (33) country: Spain	Retrospective cohort study	A total of 2,850 women were classified on basis of basis of P level into following groups: <0.5 ng/ml (<p10), 0.50–0.70 ng/ml, (p10–p25), 0.71–1.00 ng/ml (p25–p50), 1.01–1.40 ng/ml (p50–p75), 1.41–1.80 ng/ml (p75–p90), and >1.81 ng/ml (>p90)	CPR and implantation rate was assessed on the basis of five distinct serum P levels	P levels neither had a negative impact on the oocyte quality and endometrial receptivity nor did it affect pregnancy success. Only in the group of P level >1.80 ng/ml there was a marginally significant negative impact on pregnancy rates (OR: 0.73, 95% CI: 0.61–0.99)

Cruz et al. (33) country: Spain	Retrospective study	A retrospective analysis of 1,800 cycles comparing high (>1.5 ng/ml) or low (<1.5 ng/ml) progesterone levels in patients undergoing controlled stimulation classified as high responders (E2 > 3,000 pg/ml) was done	The study aimed to determine the influence of high progesterone levels on clinical outcomes in high ovarian response	There was no significant differences in the analyzed parameters, namely, number of retrieved oocytes (17.2 ± 0.8 versus 17.3 ± 0.4), number of transferred embryos (1.81 ± 0.08 versus 1.85 ± 0.02), pregnancy rate (59.9 versus 54.6%), implantation rate (41.2 versus 39.7%), and miscarriage rate (22.6 versus 28.6%) for high and low progesterone levels, respectively in case of high ovarian response

*IVF, in vitro fertilization; LBR, live birth rates; RCT, randomized controlled trials; hMG, human menopausal gonadotropin; rhFSH, recombinant human follicle stimulating hormone; CPR, clinical pregnancy rates; FET, frozen-embryo transfer; GnRha, gonadotropin releasing hormone agonist; P, progesterone; OPR, ongoing pregnancy rate; ET, embryo transfer; hCG, human chorionic gonadotropin*.

### Which P Thresholds Are Best to Identify Patients at Risk of Implantation Failure due to PE in a Fresh Embryo Transfer?

Some studies have examined the impact of different P thresholds on fresh embryo transfer IVF/ICSI pregnancy outcomes. In one study, Xu et al. assessed the effect of PE on the day of hCG on pregnancy following fresh and frozen embryo transfer (FET) as a function of the ovarian response to COS: high (≥20 oocytes; *n* = 2,023), poor (≤4 oocytes; *n* = 27), or intermediate (*n* = 8,205) (14). The P cutoff points associated with decreased pregnancy outcomes in fresh embryo transfer cycles were 1.5 ng/ml for poor responders, 1.75 ng/ml for intermediate responders, and 2.75 ng/ml for high responders. The authors also observed that P mean values differed according to ovarian response (1.89 ± 0.66 ng/ml in high responders, 1.47 ± 0.47 ng/ml in intermediate responders, and 1.18 ± 0.48 ng/ml in poor responders, *p* < 0.001), with PE being more common in the high ovarian response group than intermediate and poor ovarian response groups.

Furthermore, whereas OPRs were negatively affected by PE in fresh embryo transfer IVF/ICSI cycles, no detrimental effect was reported in the FET cycles. A multivariate logistic regression analysis showed that the rise in P level was associated with the number of oocytes retrieved, FSH dose, and estradiol values on the day of HCG administration. Last, the authors found that the detrimental effect of PE on the implantation rate and OPR were independent of oocyte quality (14).

In another study, Venetis et al. retrospectively analyzed 3,296 fresh IVF/ICSI cycles to assess the effect of PE on live birth rates (LBR) (13). A bivariate analysis reported no statistical difference in the LBRs when patients with normal *P* values (<1.5 ng/ml) were compared to patients with PE (≥1.5 ng/ml; OR: 0.78, 95% confidence interval (CI): 0.56–1.09). However, a multivariable regression analysis showed an overall decrease in LBR in the elevated P level group (OR: 0.68, 95% CI: 0.48–0.97). The study also assessed whether the effect of PE on LBR was associated with the ovarian response. There was no statistically significant impact of PE on LBRs in both low (<6 oocytes, *n* = 796 cycles) and high (>18 oocytes, *n* = 730 cycles) ovarian response groups, whereas negative effect of PE was reported in normal responders (6–18 oocytes, *n* = 1,770 cycles) with threshold levels as low as 0.9 ng/ml being significant. The authors reported that the number of oocytes and female age were the most common confounding factors for PE (13).

In a recent cohort study performed by Shufaro et al., the impact of late follicular phase PE was also assessed as a function of ovarian response to COS. The authors included 8,649 IVF/ICSI cycles performed in women aged 33.9 ± 5.8 years in whom pituitary suppression was used (GnRH agonist: 49.8% of cycles; GnRH antagonist: 50.2% of cycles). The mean number of follicles >14 mm in diameter recorded before hCG administration was 8.07 ± 3.31 (range: 3–15 follicles). In addition to measure late follicular phase P levels, the authors determined the progesterone-to-follicle index (PFI), which was considered to be more representative of the intrinsic follicular properties that are related to cycle outcome than the total blood P. The PFI was calculated by dividing the blood P by the number of follicles ≥14 mm. The CPR was calculated against the range of PFI values and serum P levels. Overall, the mean late follicular phase P level was 2.22 ± 1.33 nmol/l, and the mean PFI was 0.32 ± 0.25 nmol/l follicle on the day of hCG. The (reverse) ORs for pregnancy after a fresh embryo transfer were 1.11 [95% confidence interval (CI): 1.07–1.16] for serum P and 4.10 (95% CI: 3.18–5.28) for the PFI. These authors reported that elevated P levels were associated with a lower pregnancy rate only when they reached the >93rd percentile. In contrast, the PFI was inversely and linearly related to the pregnancy rate for the whole range of values. The authors concluded that a late increase in P level was detrimental if it resulted from increased P production per follicle (high PFI), but not if it caused by additional follicular recruitment (45).

Last, recent reports have examined the issue from a different perspective. In one study, Lee et al. assessed the effect of the duration of preovulatory PE on pregnancy rates (15). Persistence of PE for 2 days or more was significantly associated with a reduction in the CPR (39.4 versus 20.7%; *p* < 0.001). The overall incidence of PE was 28.4% (*n* = 173 of 610). Among them, 83.2% (*n* = 144) had 1 day of PE, 12.7% (*n* = 22) had 2 days, and 4.1% (*n* = 7) had 3 days of PE. The serum P concentration on the LH surge day in those women without PE was 3.2 ± 1.0 nmol/l (0.9–4.9) [mean ± SD (range)], whereas in those with PE it was 6.4 ± 1.7 nmol/l (5.0–15.3); the serum E2 concentration was 1,082 ± 329 pmol/l (376–2,222) and 1,045 ± 428 pmol/l (440–2,649), respectively. There were no significant differences in both clinical and ongoing PR (39.0 versus 37.3% and 32.5 versus 31.7%) between those with and without PE on the day of LH surge. The incidence of PE in frozen-thawed embryo transfers in subsequent natural cycles (FET-NC) was not significantly different than that in stimulated cycles (15). In another study, Lai et al. evaluated the relationship between serum levels of P and estradiol (P:E ratio) measured on the day of hCG administration on ART clinical outcomes of 139 infertile women with normal ovarian reserve treated with a long GnRH-a protocol. The authors showed that P:E ratio was significantly higher among patients with premature luteinization (PL, *n* = 41), defined by a P:E ≥ 1.2 using receiver operator characteristic analysis, than in non-PL (*n* = 98) group (2.4 ± 1.5 and 0.6 ± 0.3, respectively), but P:E ratio did not correlate with pregnancy outcomes after a fresh embryo transfer (46).

A critical aspect to discuss about P thresholds concerns the shortcomings of the currently rapid immunoassays for steroid determination. These assays have been associated with poorly agreeable results due to low assay specificity, poor optimization of methods over the large concentration ranges observed clinically, and inadequate standardization (47, 48). Although direct immunoassay platforms are fully automated, easy-to-use, inexpensive, and allow rapid detection with a high throughput, there is limited data regarding the performance and precision of these immunoassay systems, particularly in the lower range of detectable P concentrations (<2.5 ng/ml) (48). For automated immunoassay platforms, intra-assay variability is generally expressed as an averaged variability across discrete analyte standards spanning low, middle, and high ranges of P levels. It has been argued that these systems should not be used clinically for low detection limits such as measuring P with values corresponding to ~1 ng/ml. Due to the non-uniform sensitivity of P measurement assays, especially in the low range of P, thus, caution should be applied to adopt any P threshold level for managing purposes, such as the recommendation of freezing all embryos in cycles with P elevation on day of hCG administration based on a single measurement and using specific low range P cutoff points (49).

Collectively, these observations indicate that there is still no clarity on the cutoff value of P above which it negatively affects pregnancy success in fresh embryo transfer IVF/ICSI cycles. Alternative approaches including the determination of PE duration and measurement of P to follicle index ratio (PF) may prove useful to assess the role of follicular phase PE on pregnancy success, but further research is needed to confirm their clinical utility.

### Should a “Freeze-All” Policy be Adopted in All the Cycles With PE on the Day of hCG?

Based on the existing literature, it is clear that the association between PE and IVF/ICSI success does not follow a binomial distribution, i.e., PE not always halt implantation regardless of the threshold level adopted. Therefore, the next critical question for clinicians would be to know the absolute pregnancy reduction rate in the face of PE.

According to the largest systematic review and meta-analysis published to date, the OR for pregnancy reduction associated with PE was 0.64 (95% CI: 0.54–0.76). Transformation of the OR mentioned above into absolute pregnancy rate reduction (APRR) translated in 10.1% APRR (95% CI: 6–14%) (12). Using these assumptions, it is possible to estimate the net effect of performing fresh embryo transfer in cases of PE for a given IVF Program. In Table 3, we estimated the net effect of PE on pregnancy for an IVF center performing 1,000 cycles per year with an overall baseline pregnancy rate of 40% per fresh embryo transfer, considering three different scenarios of PE incidence as commonly reported in the literature. According to these estimations, the net effect on pregnancy reduction for the overall population subjected to fresh embryo transfer IVF/ICSI cycles in a given IVF center would range from 0.5 to 3.0% in the best and worst case scenarios, respectively. It means clinicians in that hypothetical center would need to monitor P levels in 1,000 cycles and intervene in 50–300 cycles with PE to potentially avoid 2–12 implantation failures by applying the freeze-all strategy. Notably, despite improvements in cryopreservation techniques and an overall favorable outcome with the transfer of frozen-thawed vitrified embryos (42, 50, 51), pregnancy rates reported by individual centers still vary, with an success rate of approximately 50% (52). As a result, the final net effect of PE on pregnancy rates may be offset even further.

**Table 3:** Different scenarios to evaluate net effect of PE on pregnancy for an IVF center performing 1,000 cycles per year.

Cycles	Scenario 1	Scenario 2	Scenario 3
Cycles with PE (%)	5	15	30
Cycles with PE^a^ (*N*)	50	150	300
Expected pregnancies in the subgroup of PE^b^ (*N*)	20	60	120
Achieved pregnancies corrected by APRR^c^ (*N*)	18	54	108
Overall pregnancy reduction per 1,000 cycles; *N* (%)	2 (0.5%)^d^	6 (1.5%)^d^	12 (3.0%)^d^

*^a^Per 1,000 cycles*.

*^b^Considering 40% as the overall PR per fresh embryo transfer*.

*^c^Considering 10% absolute pregnancy rate reduction (APRR)*.

*^d^400 pregnancies would be expected overall per 1,000 cycles*.

Thus, fresh embryo transfers in IVF/ICSI cycles with PE should not be disregarded, particularly when supranumerary embryos will make cryopreservation inevitable. Furthermore, recent evidence indicates that individualization of the luteal phase support by addition of LH activity to progesterone supplementation for fresh ET can overcome the luteal phase deficiency in infertile patients submitted to IVF/ICSI cycles with GnRH antagonist cotreatment who underwent fresh embryo transfer. There are still concerns that the adoption of a universal segmentation policy for PE might compromise the overall health of the mother and generated offspring (53). There have been reports of increased incidence of placenta accreta, preeclampsia, macrosomia, and large for gestational age as a result of the transfer of frozen-thawed embryos compared to fresh counterparts (54). Along the same lines, adoption of a freeze-all policy would require additional embryo manipulation that might induce epigenetic changes, and thus further add risk to children born from frozen-thawed embryo transfer cycles (55–61).

In conclusion, the current limitations of progesterone assays, the conflicting data on P thresholds, and the equivocal clinical implications of PE to pregnancy outcomes should prompt clinicians to critically evaluate their dataset and practices to determine the usefulness of routine measurement of progesterone levels on the day of hCG trigger and adopting a freeze-all strategy to all cycles of PE. At present, the indiscriminate adoption of 1.5 ng/ml cut off to adopt an embryo freeze-all strategy is not evidence-based. Moreover, the net effect of PE on pregnancy success of ART units is likely minimal.

## Future Aspects

An analysis of the freeze all policy adopted in all the cycles with PE on the day of hCG, taking into account cost-effectiveness, patient-centeredness, and time to live-birth has to be carried out to better assess the clinical validity of such additional interventions. Furthermore, development of robust assays to measure P at low limit levels is essential to determine PE cutoff levels with clinical impact. Better identification of the patient profile at higher risk of implantation failure due to PE on the late follicular phase and the optimal algorithm for PE determination is of utmost importance for clinicians providing care to infertile couples undergoing ART.

## Conclusion

Conflicting evidence still prevails concerning the effect of P elevation on the day of hCG trigger in fresh embryo transfer IVF/ICSI pregnancy outcomes. Clinicians should exercise caution to adopt cutoff values of P in the clinical management of patients with PE due to limitations of exiting assays to measure progesterone at the lower levels and the lack of unequivocal evidence indicating a negative effect of PE on pregnancy success in fresh embryo transfer IVF/ICSI cycles. Gonadotropin type and regimen, as routinely used during COS, seems to have an eligible effect on late follicular phase progesterone levels. However, given the higher potency of recombinant gonadotropin preparations than urinary counterparts and the possibility of small dose adjustments using the former, individualized dose adaptation can be used during COS to modulate the GCs progesterone output. A potential adverse impact of PE on pregnancy success does not seem to affect the overall patient population equally, with high responders to COS being less susceptible to the possible detrimental effect of PE. An individualized approach should be used in cases of PE, which could include fresh embryo transfers in hyper-responders with low risk of OHSS and in patients with supranumerary embryos undergoing blastocyst transfer. In normal responders with PE undergoing day 3 fresh embryo transfers, a “freeze-all” strategy might be considered. As for poor responders, the optimal strategy in the face of PE is yet to be determined.

## Authors Contributions

GK, SG, DR, SD, KI, MP, JS, KM, and KM were involved in critical analysis of data and provided expert comments and amendments for formulation of the manuscript. PT participated in its design and coordination, primarily searched the literature, and helped to draft the manuscript. PD organized the meetings and helped to formulate the study questions, participated in its design and coordination, primarily searched the literature, and helped to draft the manuscript. SE designed the review, helped in coordination and literature search, provided critical analysis of data, and drafted the manuscript. All authors approved its final version.

## Conflict of Interest Statement

Authors PD and PT were both employed by company Merck Ltd., India. The funders had no role in study design, data collection and analysis, decision to publish, or preparation of the manuscript. All authors declare no conflict of interest. The reviewer AC and handling Editor declared their shared affiliation.
