# Supplementary material for: Association Between Progesterone Elevation on the Day of Human Chronic Gonadotropin Trigger and Pregnancy Outcomes After Fresh Embryo Transfer in In Vitro Fertilization/Intracytoplasmic Sperm Injection Cycles
Source: Front Endocrinol (Lausanne). 2018 Apr 26;9:201. doi: 10.3389/fendo.2018.00201 (PMC5932157; doi:10.3389/fendo.2018.00201)
Supplement: Supplementary file 1 [file data_sheet_1.DOCX]

**Supplementary Table:**

**List of Studies used to Address the Clinical Questions Concerning the Role of Progesterone Elevation of ART Pregnancy Outcomes.**

| **S.No** | **Author and Year** | **Study Title** | **Study Design and**  **Level of Evidence** | **Clinical question** |
| --- | --- | --- | --- | --- |
| 1 | Anderson *et al.* 2011 | Preovulatory progesterone concentration associates signiﬁcantly to follicle numberand LH concentration but not to pregnancy rate | Multicentre, a large prospective randomized controlled trial  Ib and IIb | 1 and 2 |
| 2 | Griesinger *et al.* 2013 | Progesterone elevation does not compromise pregnancy rates in high responders: a pooled analysis of in vitro fertilization patients treated with recombinant follicle-stimulating hormone/gonadotropin-releasing hormone antagonist in six trials. | Retrospective combined analysis from six clinical trials.  Ia | 2 |
| 3 | Requena *et al. 2013* | High progesterone levels in women with high ovarian response do not affect clinical outcomes: a retrospective cohort study | Retrospective cohort study  III | 1 and 2 |
| 4 | Xu *et al. 2012* | Serum progesterone level effects on the outcome of in vitro fertilization in patients with different ovarian response: an analysis of more than 10,000 cycles. | Noninterventional, retrospective, single-center cohort  study  III | 3 |
| 5 | Venetis *et al.* 2015 | Estimating the net effect of progesterone elevation on the day of hCG on live birth rates after IVF: a cohort analysis of 3296 IVF cycles. | Retrospective cohort study at single centre  III | 3 |
| 6 | Shufaro *et al.* 2015 | Progesterone-to-follicle index is better correlated with in vitro fertilization cycle outcome than blood progesterone level. | Cohort study  III | 3 |
| 7 | Roque*et al.* 2015 | Freeze-All policy: fresh vsfrozen-thawed embryo transfer | Prospective, observational cohort study  III | 3 and 4 |
| 8 | SverreBjercke*et al.* 2010 | Clinical outcome following stimulation with highly purified hMG or recombinant FSH in patients undergoing their first treatment cycle of IVF or ICSI | Prospective cohort study  III | 1 |
| 9 | Devroey*et al*.2012 | A randomized assessor-blind trialcomparing highly puriﬁed hMGand recombinant FSH in a GnRHantagonist cycle with compulsorysingle-blastocyst transfer | Randomized, open-label, assessor-blind, parallel groups, multicenter, noninferiority trial.  Ib and IIb | 1 |
| 10 | Devroey*et al*. 2009 | A double-blind, non-inferiority RCT comparing corifollitropinalfa and recombinant FSH during the first seven days of ovarian stimulation using a GnRH antagonist protocol. Human reproduction. | Large, double-blind, randomized, non-inferiority trial  Ib and IIb | 1 |
| 11 | Bosch *et al.* 2010 | Circulating progesterone levels and ongoing pregnancy rates in controlled ovarian stimulation cycles for in vitro fertilization: analysis of over 4000 cycles. | A non-interventional, retrospective, observational, single-centre cohort study  III | 2 |
| 12 | Cruz *et al.* 2013 | High progesterone levels in high ovarian response do not affect  Clinical outcomes. | Retrospective cohort study  III | 2 |
| 13 | Lee*et al.* 2014 | Effect of preovulatory progesterone elevation and duration of progesterone elevation on the pregnancy rate of frozen–thawed embryo transfer in natural cycles | Retrospective cohort study  III | 3 |
| 14 | Lawrenz*et al.*2016 | Impact of gonadotropin type on progesterone elevation during ovarian stimulation in GnRH antagonist cycles | Metaanalysis of two multi-center, randomized, double-blind, double-dummy, active-controlled, non-inferiority trials  Ia and IIb | 1 |
| 15 | Papanikolaou*et al.* 2009 | Progesterone rise on the day of human chorionic gonadotropin administration impairs pregnancy outcome in day 3 single-embryo transfer, while has no effect on day 5 single blastocyst transfer | Prospective cohort study  III | 2 |
| 16 | Kolibianakis*et al.* 2012 | Significantly lower pregnancy rates in the presence of progesterone elevation in patients treated with GnRH antagonists and gonadotrophins: a systematic review and meta-analysis | Systematic review and meta-analysis  I | 1 |
| 17 | Hamdine*et al.* 2014 | Elevated early follicular progesterone levels and in vitro fertilization outcomes: a prospective intervention study and meta-analysis | Multicenter randomized controlled trial and a systematic review and meta-analysis.  Ia and IIb | 2 |
| 18 | Lee *et al.* 2014 | Effect of preovulatory progesterone elevation and duration of progesterone elevation on the pregnancy rate of frozen-thawed embryo transfer in natural cycles | Retrospective cohort study  III | 3 |
| 19 | Li *et al.* 2008 | Serum progesterone concentration on day of hCG administration and IVF outcome. | Prospective cohort study  III | 2 |
| 20 | Lai *et al.* 2009 | An increased serum progesterone-to-estradiol ratio on the day of human chorionic gonadotropin administration does not have a negative impact on clinical pregnancy rate in women with normal ovarian reserve treated with a long gonadotropin releasing hormone agonist protocol. | Retrospective cohort study  III | 2 |
| 21 | Yding Andersen *et al.* 2011 | Preovulatory progesterone concentration associates significantly to follicle number and LH concentration but not to pregnancy rate | A large, prospective randomized controlled trial  Ib, IIb | 1 |
| 22 | Eleno*et al.* 2006 | Predictive value of plasma progesterone on hCG administration day in the outcome of IVF-ET cyles. | Prospective cohort study  III | 1 |
| 23 | Bosch *et al.* 2003 | Premature luteinization during gonadotropin-releasing hormone antagonist cycles and its relationship with in vitro fertilization outcome. Fertility and sterility. | Prospective cohort study  III | 2 |
| 24 | Lee *et al.*2013 | Luteal phase support does not improve the clinical pregnancy rate of natural cycle frozen-thawed embryo transfer: a retrospective analysis. | Review article | 3 |
| 25 | Humaidan*et al.* 2014 | A review of luteinizing hormone and human chorionic gonadotropin when used in assisted reproductive technology. | Review article | 1 |
| 26 | Wennerholm*et al.* 2013 | Perinatal outcomes of children born after frozen-thawed embryo transfer: a Nordic cohort study from the CoNARTaS group. | Retrospective cohort study  III | 3 |
| 27 | Ishihara *et al.*2014 | Impact of frozen-thawed single-blastocyst transfer on maternal and neonatal outcome: an analysis of 277,042 single-embryo transfer cycles from 2008 to 2010 in Japan. | Retrospective cohort study  III | 3 |
| 28 | Kaser*et al.*2015 | Cryopreserved embryo transfer is an independent risk factor for placenta accreta | Case control study  III | 3 and 4 |
| 29 | Thuesen*et al*. 2012 | A randomized controlled dose–response pilot study of addition of hCG to recombinant FSH during controlled ovarian stimulation for in vitro fertilization. Human reproduction.. | A prospective randomized, controlled, open-label dose–response pilot study  Ib and IIb | 1 |
| 30 | Seow*et al.* 2007 | Subtle progesterone rise in the single-dose gonadotropin-releasing hormone antagonist (cetrorelix) stimulation protocol in patients undergoing in vitro fertilization or intracytoplasmic sperm injection cycles. | Prospective observational cohort study  III | 1 |
| 31 | Shapiro *et al.*2012 | Evidence of impaired endometrial receptivity after ovarian stimulation for in vitro fertilization: a prospective randomized trial comparing fresh and frozen–thawed embryo transfer in normal responders. | Randomized controlled trial  Ib and IIb | 1 |

**Clinical Questions**: #1:Is gonadotropin type associated with PE during the follicular phase of stimulated cycles?;#2: Is PE on the day of hCG associated with negative fresh embryo transfer IVF/ICSI outcomes in all patient subgroups?; #3: Which P thresholds are best to identify patients at risk of implantation failure due to PE in a fresh embryo transfer?, and #4: Should a freeze all policy be adopted in all the cycles with PE on the day of hCG?

**Levels of Evidence**: Evidence obtained from- # Level 1. Systematic reviews/ Ia- Metaanalysis of RCTs ,Ib- at least one RCT # Level 2. IIa- Well designed controlled studies without randomization and IIb: well designed at least one quasi - experimental studies # Level 3. Well designed non experimental descriptive, comparative and correlation , cohort case control studies or case series, # Level 4. Expert opinion or committee reports
